# Cancer history is an independent risk factor for mortality in hospitalized COVID-19 patients: a propensity score-matched analysis

**DOI:** 10.1186/s13045-020-00907-0

**Published:** 2020-06-10

**Authors:** Yifan Meng, Wanrong Lu, Ensong Guo, Jia Liu, Bin Yang, Ping Wu, Shitong Lin, Ting Peng, Yu Fu, Fuxia Li, Zizhuo Wang, Yuan Li, Rourou Xiao, Chen Liu, Yuhan Huang, Funian Lu, Xue Wu, Lixin You, Ding Ma, Chaoyang Sun, Peng Wu, Gang Chen

**Affiliations:** 1 National Clinical Research Center of Gynecology and Obstetrics, Tongji Hospital, Tongji Medical College, Huazhong University of Science and Technology, Wuhan, 430030 China; 2 Cancer Biology Research Center, Tongji Hospital, Tongji Medical College, Huazhong University of Science and Technology, Wuhan, 430030 China

**Keywords:** COVID-19, SARS-CoV-2, Cancer history, Independent risk factor, Mortality, Comorbidities

## Abstract

**Background:**

Although research on the effects of comorbidities on coronavirus disease 2019 (COVID-19) patients is increasing, the risk of cancer history has not been evaluated for the mortality of patients with COVID-19.

**Methods:**

In this retrospective study, we included 3232 patients with pathogen-confirmed COVID-19 who were hospitalized between January 18th and March 27th, 2020, at Tongji Hospital in Wuhan, China. Propensity score matching was used to minimize selection bias.

**Results:**

In total, 2665 patients with complete clinical outcomes were analyzed. The impacts of age, sex, and comorbidities were evaluated separately using binary logistic regression analysis. The results showed that age, sex, and cancer history are independent risk factors for mortality in hospitalized COVID-19 patients. COVID-19 patients with cancer exhibited a significant increase in mortality rate (29.4% vs. 10.2%, *P* < 0.0001). Furthermore, the clinical outcomes of patients with hematological malignancies were worse, with a mortality rate twice that of patients with solid tumors (50% vs. 26.1%). Importantly, cancer patients with complications had a significantly higher risk of poor outcomes. One hundred nine cancer patients were matched to noncancer controls in a 1:3 ratio by propensity score matching. After propensity score matching, the cancer patients still had a higher risk of mortality than the matched noncancer patients (odds ratio (OR) 2.98, 95% confidence interval (95% CI) 1.76–5.06). Additionally, elevations in ferritin, high-sensitivity C-reactive protein, erythrocyte sedimentation rate, procalcitonin, prothrombin time, interleukin-2 (IL-2) receptor, and interleukin-6 (IL-6) were observed in cancer patients.

**Conclusions:**

We evaluated prognostic factors with epidemiological analysis and highlighted a higher risk of mortality for cancer patients with COVID-19. Importantly, cancer history was the only independent risk factor for COVID-19 among common comorbidities, while other comorbidities may act through other factors. Moreover, several laboratory parameters were significantly different between cancer patients and matched noncancer patients, which may indicate specific immune and inflammatory reactions in COVID-19 patients with cancer.

## Introduction

Severe acute respiratory syndrome coronavirus 2 (SARS-CoV-2) can cause the infectious respiratory illness known as coronavirus disease 2019 (COVID-19), which was first identified in December 2019 and has since spread globally [1–3]. As of May 16, 2020, there have been more than 4,500,000 confirmed cases worldwide. While the majority of patients experience mild symptoms, some patients may progress to pneumonia, multiorgan failure, or even death. To date, more than 300,000 patients have died from COVID-19. The overall mortality rate of diagnosed cases is 3.4%, [4] ranging from 0.2 to 22.7%, depending on age group and other health problems [5, 6]. It has been reported that the majority of those who die of COVID-19 have pre-existing conditions, including cancer, hypertension, diabetes, and cardiovascular disease [5]. Although research on the effects of comorbidities in COVID-19 patients is increasing, most related studies have reported the unadjusted risk of comorbidities with a relatively small sample size [7–10]. Age, comorbidities, lymphocytopenia, and specific elevated laboratory parameters were reported to be associated with intensive care unit (ICU) admission [7–9]. Moreover, several published works with relatively small sample sizes have reported the risk factors for mortality in patients with COVID-19 [7, 11–13]. However, studies focusing on the association of cancer history with mortality in hospitalized patients with COVID-19 are rare. To evaluate the survival outcomes and prognostic factors in cancer patients with COVID-19, we conducted a single-center retrospective analysis in Tongji Hospital, a designated hospital for severe COVID-19 patients in Wuhan, China. The strengths of our study include a large sample size and accurate baseline and complete clinical outcome data.

## Patients and methods

### Study design and participants

**Fig. 1:**
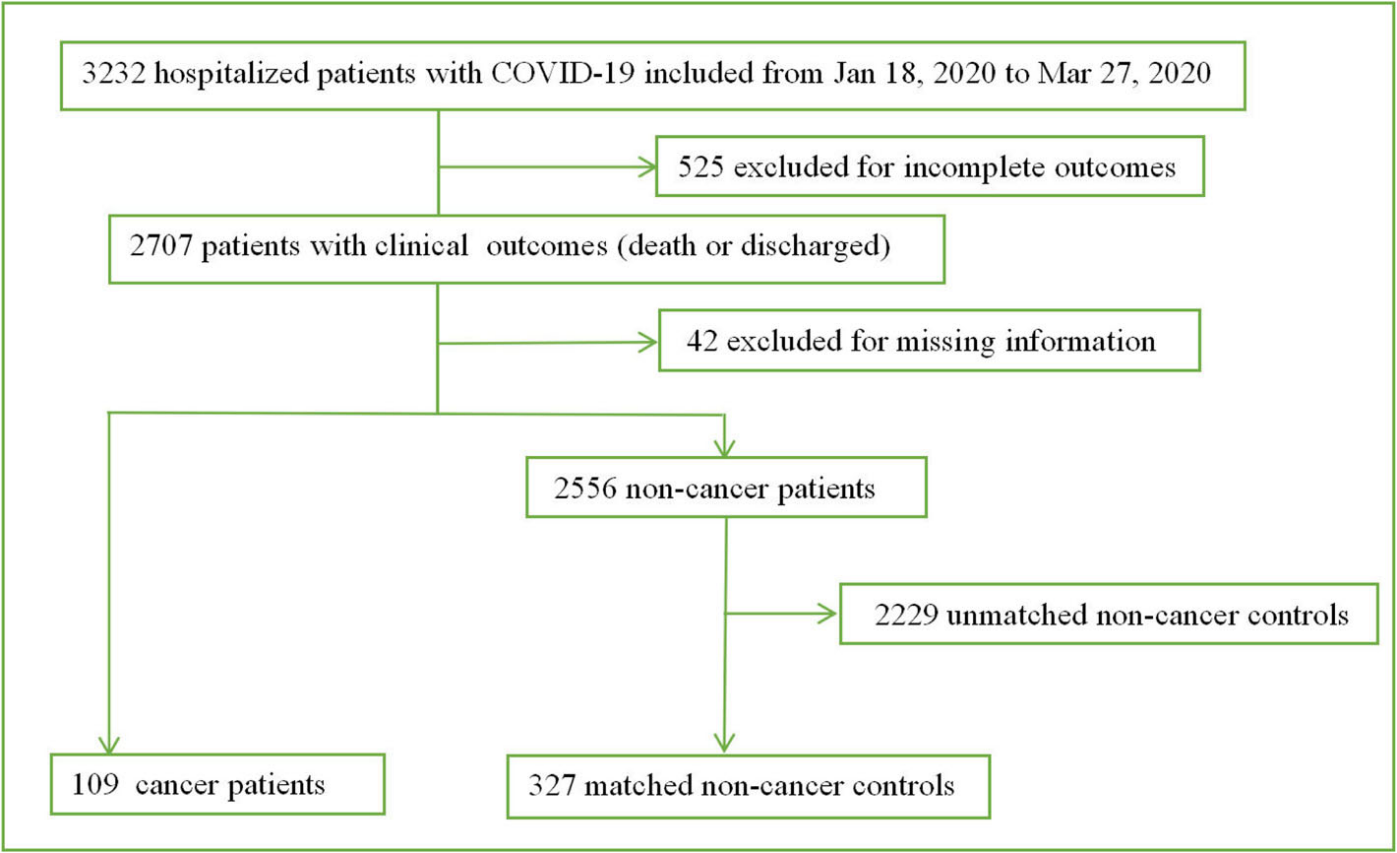
Flowchart for the inclusion of patients

In this retrospective study, we included 3232 consecutive patients with COVID-19 who were hospitalized between January 18 and March 27, 2020, at Tongji Hospital in Wuhan, China. All patients included in the present study were diagnosed with moderate to severe pathogen-confirmed COVID-19. As of March 27, 2020, 299 of the 3232 patients had died, and 2408 patients had recovered and been discharged. The remaining 525 patients were still in the hospital receiving medical care. After excluding these 525 patients with incomplete outcomes and 42 patients with missing information, a total of 2665 patients (82.5%, 2665/3232) with complete follow-up data reached the endpoints of observation (died or discharged from the hospital). The diagram of patient participation is shown in Fig. 1. This study was approved by the Ethical Committee of Tongji Hospital of Tongji Medical College at Huazhong University of Science and Technology. Written informed consent was not obtained because the data were analyzed retrospectively and anonymously.

### Procedures

Medical records were reviewed retrospectively for the following information: patient characteristics, physical examination, laboratory data, radiologic findings, treatments, and clinical outcomes. The demographic and clinical characteristics were systematically collected up to March 27, 2020, the final date of follow-up. All patients were identified according to the latest diagnosis and treatment protocol for COVID-19 (trial version 7) issued by the General Office of the National Health Commission. Symptomatic patients were verified as positive for SARS-CoV-2 infection by oropharyngeal or nasopharyngeal swabs. The specific operation methods for SARS-CoV-2 RNA with real-time reverse transcriptase polymerase chain reaction (RT-PCR) were followed as previously described [14, 15]. In the present study, the primary clinical endpoints were defined as death or discharge from the hospital. In clinical practice, patients meeting the following criteria could be discharged: (a) afebrile for > 3 days, (b) improved respiratory symptoms, (c) pulmonary imaging showing visible absorption of inflammation, and (d) nucleic acid tests negative for respiratory tract pathogens twice consecutively (sampling interval ≥ 24 h).

### Statistical analysis

Analyses were conducted using SAS version 9.4 (SAS Inc.) and R version 3.6.2 (R Foundation for Statistical Computing). The measurement data are expressed as the mean and standard deviation (SD) or median and interquartile range (IQR). The enumeration data are summarized as frequencies and percentages. The clinical endpoints were compared between cancer patients and noncancer patients using propensity score (PS) matching. We performed PS matching using a 1:3 ratio with the nearest neighbor matching procedure without replacement. The comparison between the two groups was performed by independent group *t* tests when the measurement data were normally distributed; otherwise, Mann-Whitney *U* tests were applied. Intergroup comparisons of the enumeration data were performed using chi-squared tests or Fisher’s exact tests. Univariate and multivariate logistic analyses were adopted to explore the risk factors for COVID-19 patients with cancer. Kaplan-Meier curves were plotted for the length of hospital stay for discharged patients and the time from admission to death for patients who died. The difference was assessed using log-rank tests. *P* < 0.05 was considered statistically significant.

## Results

### Baseline clinical characteristics and assessment of risk factors for mortality

From January 18, 2020, to March 27, 2020, 3232 hospitalized patients with COVID-19 were included in the present study. After necessary exclusion, 2665 patients were included in our analysis (Fig. 1). Of these patients, 293 patients died from COVID-19, and 2372 patients were discharged from the hospital. The impacts of age, sex, and comorbidities were evaluated separately using binary logistic regression analysis (Additional file 1: Table S1). Significant factors that increased the risk of mortality were male sex, older age, and cancer history (*P* < 0.0001). For other comorbidities, the results showed that hypertension, coronary heart disease (CHD), chronic obstructive pulmonary disease (COPD), and cerebrovascular disease were risk factors for mortality (*P* = 0.0005, 0.0037, 0.0008, and 0.0003, respectively), while the effects were nonsignificant after adjustment (*P* > 0.05).

### Assessment of risk factors for mortality in 109 COVID-19-infected cancer patients

Among all 109 COVID-19-infected cancer patients, eighteen patients with present cancer had received at least one kind of antitumor therapy within 3 months, such as surgery (4/109, 3.7%), chemotherapy (11/109, 10.1%), targeted therapy (1/109, 0.9%), radiotherapy (2/109, 1.8%), and immunotherapy (1/109, 0.9%); one of them received treatment combining chemotherapy and radiotherapy. In addition to cancer, 16 (14.7%) patients had more than one comorbidity. The most common comorbidities were hypertension (30/109, 27.5%), diabetes (11/109, 10.1%), and CHD (11/109, 10.1%). Table 1 displays the univariate analysis of risk factors for mortality in 109 COVID-19-infected cancer patients. The number of complications was also evaluated in these patients. The results indicated a significantly increased risk of mortality in cancer patients with complications (OR 16.80, 95% CI 3.81–74.19), while patients with at least two complications had a significantly higher risk of poor outcomes (OR 110.25, 95% CI 22.79–533.29). Cancer cachexia has been defined as a loss of lean tissue mass or as BMI < 20 kg/m^2^. In the present analysis, an odds ratio (OR) of 2.40 [95% confidence interval (95% CI) 0.44–12.98] was observed for cancer patients with BMI < 20 kg/m^2^. All cancer patients were classified according to the time from cancer diagnosis to hospitalization (< 1 year, 1–4 years, 5–9 years, > 10 years). In our analysis, we found a higher risk of mortality in patients who were diagnosed with cancer within 10 years than in those who had survived cancer for more than 10 years, though this difference was not statistically significant (*P* > 0.05). The clinical outcomes of patients with hematological malignancies were worse, with a mortality rate twice that of patients with solid tumors (50% vs. 26.1%, *P* = 0.06). Although not statistically significant, the difference may be of clinical significance. Table 2 shows the comparison of the clinical characteristics between patients with solid tumors and those with hematological malignancies. The age distribution indicated a higher proportion of young and middle-aged adults in COVID-19 patients with hematological malignancies (*P* = 0.0039).

**Table 1:** Univariate analysis of risk factors in 109 COVID-19 patients with cancer

Risk factors	Death (*n* = 32)	Discharged (*n* = 77)	OR	95%CI	*P* value
**Sex**					
Male	19 (31.2)	42 (68.9)	REF		
Female	13 (27.1)	35 (72.9)	0.82	0.36–1.89	0.64
**Age groups**					
≤ 49 years	7(31.8)	15(68.2)	REF		
50–64 years	8(22.9)	27(77.1)	0.64	0.19–2.01	0.46
65–79 years	10(26.3)	28(73.7)	0.77	0.24–2.42	0.65
≥ 80 years	7(50.0)	7(50.0)	2.14	0.54–8.51	0.28
**Number of comorbidities**					
0	25 (33.3)	50 (66.7)	REF		
1	4 (22.2)	14 (77.8)	0.57	0.17–1.92	0.36
> 1	3 (18.8)	13 (81.3)	0.46	0.12–1.77	0.26
**Comorbidities**(REF=No)					
Hypertension	5 (16.7)	25 (83.3)	0.39	0.13–1.12	0.080
Coronary heart disease	2 (18.2)	9 (81.8)	0.50	0.10–2.47	0.40
Diabetes	2 (18.2)	9 (81.8)	0.50	0.10–2.47	0.40
Others	1 (50.0)	1 (50.0)	2.45	0.15–40.43	0.53
**Types of tumor**					
Solid tumors	24 (26.1)	68 (73.9)	REF		
Hematological malignancies	8 (50.0)	8 (50.0)	2.83	0.96–8.38	0.06
**Lung cancer or not**					
No	21 (27.6)	55 (72.4)	REF		
Yes	3 (17.7)	14 (82.4)	0.56	0.15–2.15	0.40
**Number of complications**					
0	3 (4.6)	63 (95.5)	REF		
1	8 (44.4)	10 (55.6)	16.80	3.81–74.19	0.0002^a^
≥ 2	21 (84.0)	4 (16.0)	110.25	22.79–533.29	< 0.0001^a^
**Body mass index**					
≥ 20 kg/m^2^	5 (17.2)	24 (82.8)	REF		
< 20 kg/m^2^	3 (33.3)	6 (66.7)	2.40	0.44–12.98	0.31
**Smoking history**					
Yes	11 (22.0)	39 (78.0)	REF		
No	2 (11.1)	16 (88.9)	0.44	0.09–2.23	0.32
**The time since cancer diagnosis to hospitalization**					
< 1 year	3 (21.4)	11 (78.6)	2.18	0.19–25.00	0.53
1–4 years	6 (20.7)	23 (79.3)	2.09	0.22–20.08	0.52
5–9 years	22 (38.6)	35 (61.4)	5.03	0.59–42.97	0.14
≥ 10 years	1 (11.1)	8 (88.9)	REF		

^a^Significant at *P* < 0.05

**Table 2:** Clinical characteristics of patients with solid tumors and hematological malignancies

Characteristics	Solid tumors (*n* = 92)	Hematological malignancies (*n* = 16)	*P* value
**Sex**			0.54
Male	50 (54.3)	10 (62.5)	
Female	42 (45.7)	6 (37.5)	
**Age groups**			0.0039^a^
≤ 49 years	14 (15.2)	8 (50.0)	
50–64 years	28 (30.4)	6 (37.5)	
65–79 years	36 (39.1)	2 (12.5)	
≥ 80 years	14 (15.2)	0 (0.0)	
**Comorbidities**			
Hypertension	28 (30.4)	2 (12.5)	0.23
Coronary heart disease	11 (12.0)	0 (0.0)	0.36
Diabetes	11 (12.0)	0 (0.0)	0.36
Chronic obstructive pulmonary disease	0 (0.0)	0 (0.0)	–
Chronic kidney disease	0 (0.0)	0 (0.0)	–
Cerebrovascular disease	1 (1.1)	0 (0.0)	1.0000
Hepatitis	0 (0.0)	0 (0.0)	–
Tuberculosis	1 (1.1)	0 (0.0)	1.0000

^a^Significant at *P* < 0.05

### Propensity score-matched analysis

Compared to COVID-19 patients without a history of cancer, patients with cancer were older. The baseline patient characteristics are listed in Additional file 1: Table S2. The median age (SD) for patients with cancer and noncancer controls was 61.7 (16.1) years and 57.9 (15.9) years, respectively (*P* = 0.015). The remaining indexes were not significantly different between the different groups. Overall, this comparison demonstrated that age was not comparable between the two groups. The PS approach has gained wide popularity for balancing patients’ baseline characteristics. Considering the significant impact of older age, the 109 cancer patients were matched with noncancer controls in a 1:3 ratio by PS matching. All covariates were balanced between the groups after matching. After propensity score matching, the cancer patients still had a higher risk of mortality than the matched noncancer patients (OR 2.98, 95% CI 1.76–5.06).

### Laboratory parameters and clinical courses in COVID-19-infected cancer patients and matched noncancer controls

**Fig. 2:**
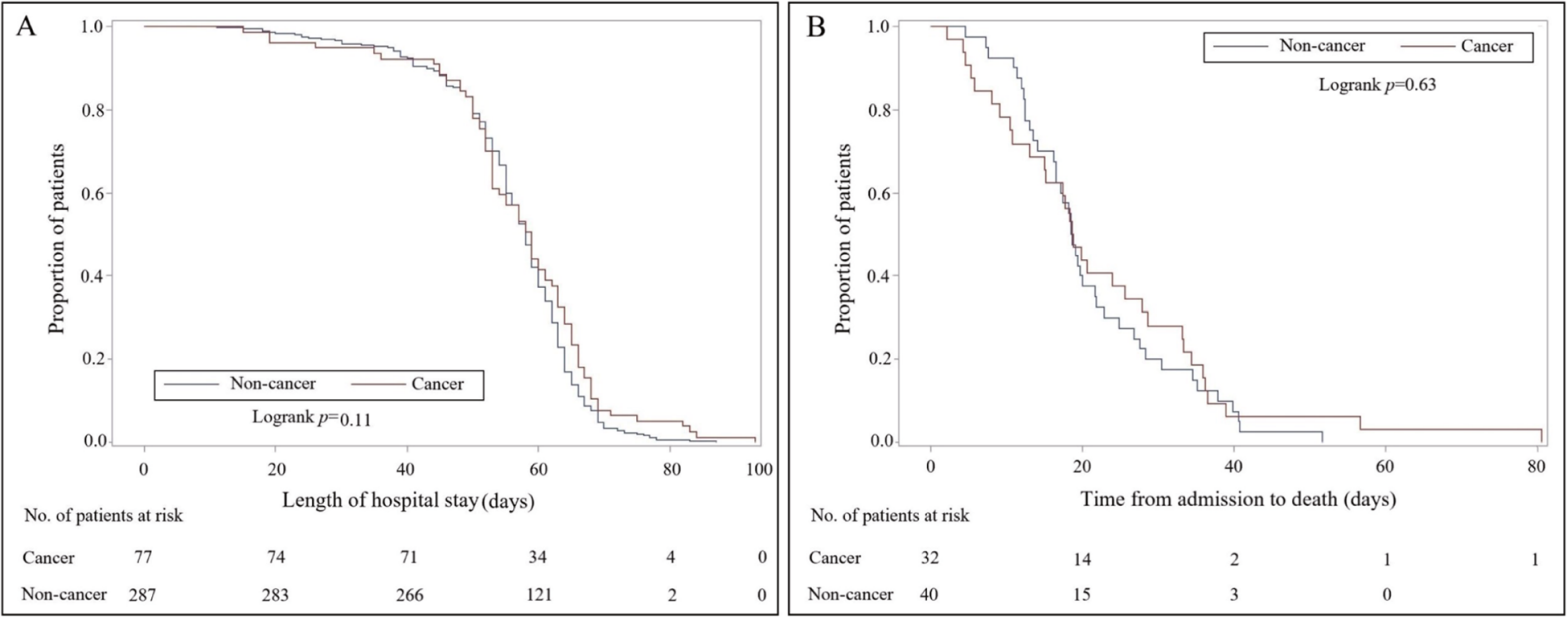
Kaplan-Meier estimates of the proportion of patients with COVID-19. **a** The length of hospital stay for discharged patients according to cancer patients and matched noncancer controls; **b** the time from admission to death for patients who died according to cancer patients and matched noncancer controls

Substantial differences in laboratory findings were observed between COVID-19-infected cancer patients and matched noncancer controls. As shown in Table 3, several laboratory parameters were significantly different between the two groups. On admission, the routine blood workup of cancer patients showed significantly lower lymphocyte counts (1.0 × 10^9^/L vs. 1.2 × 10^9^/L), hemoglobin levels (118 g/L vs. 126 g/L), and platelet counts (196.0 × 10^9^/L vs. 210.5 × 10^9^/L). Other laboratory parameters with significant differences were mainly concentrated in coagulation function indicators, inflammatory markers, and cytokines (Table 3). Figure 2a displays the Kaplan-Meier curve for the length of hospital stay for discharged patients. In the univariate (log-rank) analysis, there were no significant differences in the duration of hospitalization in COVID-19-infected cancer patients and noncancer controls (*P* = 0.11). Similarly, Fig. 2b indicates that there were no significant differences in the time from admission to death for cancer and noncancer patients who died (*P* = 0.63).

**Table 3:** Laboratory parameters, radiology, and treatments of cancer patients and matched noncancer controls

Variables	Cancer (*n* = 109)	Non-cancer (*n* = 327)	*P* value^a^
**Laboratory parameters**			
White blood cell count (×10^9^/L–median[IQR])	5.5 (4.3–7.3)	5.7 (4.6–7.4)	0.51
Neutrophil count (×10^9^/L–median[IQR])	3.9 (2.5–5.7)	3.7 (2.7–5.3)	0.88
Lymphocyte count (×10^9^/L–median[IQR])	1.0 (0.6–1.5)	1.2 (0.8–1.6)	0.0013^b^
Monocyte count (×10^9^/L–median[IQR])	0.5 (0.3–0.6)	0.5 (0.4–0.6)	0.48
Hemoglobin (g/L–median[IQR])	118 (95–130)	126 (115–137)	< 0.0001^b^
Platelet count (×10^9^/L–median[IQR])	196.0 (112.0–256.0)	210.5 (164.0–282.0)	0.0071^b^
Thrombin time (s–median[IQR])	16.1 (15.4–17.7)	16.4 (15.7–17.2)	0.33
Prothrombin time (s–median[IQR])	14.1 (13.5–14.8)	13.8 (13.2–14.3)	0.015^b^
Activated partial thromboplastin time (s–median[IQR])	40.0 (37.2–45.8)	38.2 (35.5–41.4)	0.0003^b^
Antithrombin activity (%–median[IQR])	87 (79–97)	93 (84–102)	0.024^b^
International normalized ratio of prothrombin (median[IQR])	1.1 (1.0–1.2)	1.1 (1.0–1.1)	0.016^b^
D-dimer (μg/ml–median[IQR])	1.0 (0.4–2.4)	0.7 (0.4–1.8)	0.17
Fibrinogen degradation products (μg/mL–median[IQR])	4.0 (4.0–7.4)	4.0 (4.0–6.1)	0.41
Fibrinogen (g/L–median[IQR])	4.5 (3.5–5.6)	4.3 (3.3–5.6)	0.58
Prothrombin time activity (%–median[IQR])	87 (80–96)	91 (84–99)	0.015^b^
Ferritin (ng/mL–median[IQR])	627.3 (318.7–2225.6)	474.1 (251.3–841.6)	0.0076^b^
Alanine aminotransferase (U/L–median[IQR])	19.0 (12.0–36.5)	23.0 (16.0–37.0)	0.064
Aspartate aminotransferase (U/L–median[IQR])	27 (19–39)	25 (19–37)	0.72
Albumin (g/L–median[IQR])	34.5 (30.5–38.8)	36.2 (32.0–40.3)	0.073
Total bilirubin (μmol/L–median[IQR])	9.9 (6.6–13.8)	9.5 (7–12.4)	0.36
Lactate dehydrogenase (U/L–median[IQR])	278 (195–401)	245 (199–334)	0.25
Blood urea nitrogen (mmol/L–median[IQR])	5.1 (3.4–7.9)	4.6 (3.6–5.9)	0.16
Creatinine (U/L–median[IQR])	66 (52–92)	70 (60–82)	0.26
High sensitivity C-reactive protein (mg/L–median[IQR])	37.6 (5.9–84.1)	10.8 (1.8–54.0)	0.0003^b^
Glucose (mmol/L–median[IQR])	6.0 (5.4–7.4)	5.9 (5.2–7.0)	0.75
Erythrocyte sedimentation rate (mm/h–median[IQR])	43 (16–72)	27 (14–52)	0.027^b^
Hypersensitive troponin I (pg/mL–median[IQR])	3.0 (1.9–10.4)	2.9 (1.9–8.4)	0.39
Procalcitonin (ng/mL–median[IQR])	0.09 (0.05–0.32)	0.05 (0.03–0.09)	< 0.0001^b^
Immunoglobulin A(g/L–median[IQR])	2.2 (1.5–3.0)	2.3 (1.6–2.8)	0.95
Immunoglobulin G(g/L–median[IQR])	11.1 (8.0–14.8)	10.8 (9.6–12.8)	1.00
Immunoglobulin M(g/L–median[IQR])	0.9 (0.6–1.1)	0.9 (0.7–1.2)	0.24
Complement 3 (g/L–median[IQR])	0.8 (0.7–1.0)	0.8 (0.7–0.9)	0.84
Complement 4 (g/L–median[IQR])	0.2 (0.2–0.3)	0.2 (0.2–0.3)	0.61
Interleukin 1β(pg/mL–median[IQR])	5.0 (5.0–5.5)	5.0 (5.0–5.0)	0.041^b^
Interleukin 2 receptor (U/mL–median[IQR])	666 (396–1089)	529 (348–856)	0.030^b^
Interleukin 6 (pg/mL–median[IQR])	13.80 (4.3–38.9)	5.4 (2.0–17.3)	0.0001^b^
Interleukin 8 (pg/mL–median[IQR])	11.9 (6.7–20.6)	11.6 (7.0–22.8)	0.84
Interleukin 10 (pg/mL–median[IQR])	5.0 (5.0–8.4)	5.0 (5.0–5.2)	0.0073^b^
Tumor necrosis factor α(pg/mL—median[IQR])	8.4 (6.4–11.1)	7.7 (6.1–10.0)	0.26
**Radiology (*n*[%])**			
Multiple ground glass opacities and consolidation	25 (22.9)	90 (27.5)	0.35
Bilateral involvement on chest CT scan	27 (24.8)	98 (30.0)	0.30
**Treatment (*n*[%])**			
Ventilator	26 (23.9)	36 (11.0)	0.0009^b^
Tracheal intubation	9 (8.3)	9 (2.8)	0.012^b^
Oxygen therapy	83 (76.2)	235 (71.9)	0.38

^a^*P* values comparing tumor and non-tumor patients are from *χ*^*2*^ test—Fisher’s exact test or Mann-Whitney *U* test

^b^Significant at *P* < 0.05

## Discussion

In total, 2665 COVID-19 patients with clinical outcomes were included in the present study. To the best of our knowledge, we reported the largest cohort of hospitalized patients with COVID-19. This retrospective cohort study evaluated the effects of different comorbidities and identified cancer history as an independent risk factor for mortality in COVID-19 patients. Additionally, elevations in ferritin, high-sensitivity C-reactive protein, erythrocyte sedimentation rate, procalcitonin, prothrombin time, interleukin-2 (IL-2) receptor, and interleukin-6 (IL-6) were observed in cancer patients, which may indicate specific immune and inflammatory reactions in COVID-19 patients with cancer.

An age-related decline in immune functions has been widely recognized [16]. Older age has been reported as a significant independent predictor of mortality in SARS, MERS, and COVID-19 [7, 8, 11, 17, 18]. According to the Centers for Disease Control and Prevention (CDC), people aged > 65 years account for 31% of COVID-19 infections, 45% of hospitalizations, and 80% of deaths caused by COVID-19 [19]. Chronic conditions affect significant proportions of older individuals, while specific cardiovascular drugs may upregulate the angiotensin-converting enzyme-2 receptor and elevate the risk of developing COVID-19. The present study also confirmed that increased age was associated with death in patients with COVID-19. Additionally, we also recognized sex as an independent risk factor associated with mortality, which was consistent with the findings of other studies [14, 15, 20–23]. However, it should be noted that the effects of age and sex were no longer significant among cancer patients. This anomaly indicates the overwhelming impact of cancer on the systemic immune status. The age distribution indicated a higher proportion of young and middle-aged adults in COVID-19 patients with hematological malignancies. Considering that patients with hematological malignancies appear more vulnerable to SARS-COV-2, it may explain the nonsignificant correlation between older age and mortality among all cancer patients.

To date, there are several published works focused on the risk factors for mortality in patients with COVID-19, which have reported that hypertension, diabetes, CHD, cerebrovascular disease or COPD were not independent risk factors associated with in-hospital death [7, 11]. These results corroborated the findings of our study. The current analysis showed that none of the common comorbidities were independent risk factors for mortality in hospitalized COVID-19 patients, except for cancer history. However, a meta-analysis evaluated the unadjusted risk of comorbidities in COVID-19 patients and concluded that several common underlying diseases might be risk factors for severe patients [8]. Guan et al. reported the results of an adjusted analysis and claimed that COVID-19 patients with any comorbidity had a poorer prognosis than those without [9]. It should be noted that in their study, the hazard ratios were calculated for the risk factors associated with the composite endpoints of admission to the ICU, invasive ventilation, and death. Therefore, the endpoints of observation were different from those in our study. In summary, the choice of assessing mortality rather than other endpoints may result in different conclusions between related studies.

Only a few articles have focused on the characteristics of COVID-19-infected cancer patients [10, 24–27]. In the present retrospective study, we reported the largest cohort of cancer patients. One hundred nine patients with COVID-19 had a history of cancer (4.1%, 109/2665), which is higher than the incidence of cancer in the general Chinese population (0.29%, according to 2015 estimates). The proportion was also higher than the incidence reported by a Chinese nationwide analysis of cancer patients with SARS-CoV-2 infection [24]. Considering that Tongji Hospital is a designed hospital for moderate to severe patients with COVID-19, cancer patients with COVID-19 may present a higher risk of being critically ill. Furthermore, the mortality rate of cancer patients with COVID-19 was as high as 26.4% (32/109), which was significantly higher than that in the noncancer population.

In the present study, we did not find that age, sex, specific cancer types, comorbidities, or smoking history could further increase the mortality risk of patients with cancer, which was different from our initial expectations and the findings of previous studies [26, 27]. The inconsistent conclusions may be explained by the limited sample size and inter-institutional and inter-country differences. It has been reported that the rates of ICU admission and ventilator use were higher for patients with hematological malignancies than for those with solid tumors [26, 27]. In the present study, we found that the clinical outcomes of patients with hematological malignancies were worse, with a mortality rate twice that of patients with solid tumors (50% vs. 26.1%), although the data did not show statistical significance. Therefore, we regard these findings as suggestive and believe that they should be interpreted with caution. Hematological malignancies, such as leukemia and lymphoma, can affect the immune system directly. T cell senescence and exhaustion are dominant aspects involved in immune dysfunction in hematological malignancies [28]. The dysregulation of the immune response in severe patients with COVID-19 has been emphasized, while the recruitment of immune cell populations may play a crucial role in the recovery of COVID-19 infection [28, 29].

We also found a higher risk of mortality in patients who were diagnosed with cancer within 10 years than in those who had survived cancer for more than 10 years, though this difference was not statistically significant. Patients recently diagnosed with cancer are presumably at higher risk because of the after-effects of surgery and the immunosuppressive effects of antitumor therapy, while the higher risk may also be influenced by the biological characteristics of the tumor itself, as well as the inflammatory reaction in the tumor microenvironment. The number of complications (acute respiratory distress syndrome, myocardial injury, arrhythmia, kidney injury, secondary infection, and shock) was also evaluated, and we observed a significantly increased risk of mortality in cancer patients with complications. Cancer patients with ≥ 2 complications had a significantly higher risk of poor outcomes. The associations of cardiac injury and kidney function with mortality in hospitalized patients with COVID-19 have been reported in recent publications [30–32]. Although the exact mechanisms need to be further explored, the need to consider these complications in COVID-19 management has been highlighted.

Furthermore, the 109 cancer patients were matched to noncancer controls in a 1:3 ratio by PS matching. While PS matching can result in the balance of covariates within the propensity model, the PS relies on the availability of measured covariates associated with the exposures and outcomes [33]. All covariates were balanced between the groups after matching. In logistic regression analysis, the aforementioned association between cancer history and mortality remained robust. In the propensity score-matched patients, we also comprehensively described the differences in parameter indexes between cancer and noncancer patients. Laboratory parameters with significant differences were mainly concentrated in routine blood examinations, coagulation function indicators, inflammatory markers, and cytokines. Lower lymphocyte counts were associated with immune suppression, and increased risk of infection, lymphocyte responses, and proinflammatory cytokine storms were emphasized in previous studies [34–36].

It has been reported that SARS-CoV-2 infection can be associated with coagulopathy, which is related to infection-induced inflammatory changes. We observed significant differences between cancer patients and matched noncancer controls in platelet count, prothrombin time, activated partial thromboplastin time, antithrombin activity, international normalized ratio of prothrombin, and prothrombin time activity. However, D-dimer, fibrinogen degradation products, and fibrinogen were similar between the groups, although D-dimer and consumptive coagulopathy are indicators of mortality [37, 38]. Cancer is intimately related to thrombosis. The risk of venous thromboembolism is 4- to 7-fold higher in patients with cancer than in those without cancer [39]. Considering the role of the coagulation profile in the evaluation of prognosis among COVID-19 patients, one of the reasons that cancer contributes to poor outcomes in COVID-19 patients could be the prothrombotic status, which has been confirmed by many published studies [37–39]. Further investigations are needed to clarify the relationships between prothrombotic status and general patient-related risk factors as well as other factors that are specific to a particular cancer or treatment. However, close monitoring of blood coagulation is of great importance for cancer patients with COVID-19. Early and prolonged pharmacological thromboprophylaxis with low molecular weight heparin can be considered in clinical practice [40].

Significantly elevated inflammatory markers (such as ferritin, high-sensitivity C-reactive protein, erythrocyte sedimentation rate, and procalcitonin) were also observed to be different in patients who died and patients who were discharged [41]. The clinical cytokine pattern that emerged suggested that specific immune factors were associated with disease severity, with increased plasma levels of the cytokines IL-2R, IL-6, IL-10, and TNF-α [42, 43]. In the current study, the cytokine examination of cancer patients showed significantly elevated levels of the IL-2 receptor and IL-6 in COVID-19 patients with cancer. Generally, IL-2 regulates the activities of white blood cells, while IL-6 acts as a pro-inflammatory cytokine. There is compelling evidence that the immune responses in cancer patients are active but dysfunctional [44]. Furthermore, it has been reported that the expression of cytokines is dysregulated in cancer patients, resulting in immune suppression that protects cancer cells [44]. Therefore, cancer patients have a weak immune system, which reduces their ability to fight infectious diseases. The specific pattern of cytokines may represent special immune and inflammatory reactions in COVID-19 patients with cancer. Currently, scientists have proposed utilizing IL-6 blockade to manage COVID-19-induced cytokine release syndrome. IL-6 is a prototypical protumorigenic cytokine that regulates various oncogenic processes [45]. The significantly elevated levels of IL-6 in cancer patients with COVID-19 indicated that IL-6 blockade may be effective for this specific subgroup of patients.

In the univariate (log-rank) analysis, for discharged COVID-19 patients, there were no significant differences in the duration of hospitalization between cancer patients and matched noncancer controls. Similarly, for COVID-19 patients who died, there were no significant differences in the time from admission to death between cancer and noncancer patients. Although the mortality rate was significantly higher in cancer patients, the results suggested that the clinical courses between cancer and noncancer patients were similar. The significant difference in mortality may reflect the intensity of a disease process, while similar clinical courses may reflect how the process has progressed. This phenomenon may indicate the pattern of the progression of COVID-19 infection. It is possible that the higher mortality of cancer patients is due to the course of cancer itself and not the impact of cancer on the course of COVID-19.

Several limitations should be noted in the present study. First, because of the limited data availability and emergency of the COVID-19 outbreak in this study, the design was not a multicenter prospective study. The present study was a retrospective analysis that was performed in a single institution, including only patients who were hospitalized and excluding asymptomatic or mild patients. More clinical and basic experimental studies are needed to further confirm our findings. Second, although the total sample size was relatively large, the data on the clinical characteristics and outcomes of COVID-19 patients with certain types of cancers are insufficient. Therefore, we emphasized the need for detailed analyses of each specific cancer. Finally, the long-term impact of COVID-19 on the prognosis of cancer and noncancer patients is still unclear in our study population and needs to be elucidated.

In conclusion, we evaluated prognostic factors with epidemiological analysis and highlighted a higher risk of mortality for cancer patients with COVID-19. Importantly, cancer history was the only independent risk factor for COVID-19 among common comorbidities, while other comorbidities may act through other factors. Moreover, several laboratory parameters were significantly different between cancer patients and matched noncancer patients, which may indicate specific immune and inflammatory reactions in COVID-19 patients with cancer.

## Supplementary information

**Additional file 1: Table S1.** Univariate and multivariate analysis of risk factors in 2665 included patients. **Table S2.** Baseline characteristics of cancer patients and non-cancer controls (Before PS Matching vs. After PS Matching)

## Data Availability

The datasets used and analyzed during the current study are available from the corresponding author on reasonable request.

## Abbreviations

95% CI: 95% confidence interval
CHD: Coronary heart disease
COPD: Chronic obstructive pulmonary diseases
COVID-19: Coronavirus disease 2019
ICU: Intensive care unit
IL-2: Interleukin-2
IL-6: Interleukin-6
IQR: Interquartile range
OR: Odds ratio
PS: Propensity score
Real-time RT-PCR: Real-time reverse transcriptase polymerase chain reaction
SARS-CoV-2: Severe acute respiratory syndrome coronavirus 2
SD: Standard deviation
