# Supplementary material for: Cancer history is an independent risk factor for mortality in hospitalized COVID-19 patients: a propensity score-matched analysis
Source: J Hematol Oncol. 2020 Jun 10;13:75. doi: 10.1186/s13045-020-00907-0 (PMC7286218; doi:10.1186/s13045-020-00907-0)
Supplement: Supplementary file 1 — Additional file 1: Table S1. Univariate and multivariate analysis of risk factors in 2665 included patients. Table S2. Baseline characteristics of cancer patients and non-cancer controls (Before PS Matching vs. After PS Matching) [file 13045_2020_907_MOESM1_ESM.docx]

Table S1. Univariate and multivariate analysis of risk factors in 2665 included patients

| **Risk factors** | **Death**  **(n=293)** | **Discharged**  **(n=2372)** | **Univariate analysis** | | **Multivariate analysis** | |
| --- | --- | --- | --- | --- | --- | --- |
|  |  |  | **OR(95%CI)** | ***P* value** | **OR(95%CI)** | ***P* value** |
| **Sex** |  |  |  |  |  |  |
| Male | 195(14.7) | 1133(85.3) | REF |  | REF |  |
| Female | 98(7.3) | 1239(92.7) | 0.46(0.36-0.59) | <0.0001^a^ | 0.46(0.35-0.60) | <0.0001^a^ |
| **Age groups** |  |  |  |  |  |  |
| ≤49 years | 14(2.0) | 693(98.0) | REF |  | REF |  |
| 50-64 years | 80(8.8) | 827(91.2) | 4.79(2.69-8.52) | <0.0001^a^ | 5.06(2.82-9.06) | <0.0001^a^ |
| 65-79 years | 142(16.4) | 724(83.6) | 9.70(5.55-16.96) | <0.0001^a^ | 9.97(5.64-17.63) | <0.0001^a^ |
| ≥80 years | 57(30.8) | 128(69.2) | 22.03(11.92-40.71) | <0.0001^a^ | 20.33(10.76-38.44) | <0.0001^a^ |
| **Comorbidities** |  |  | (REF=No) |  | (REF=No) |  |
| Hypertension | 111(14.4) | 661(85.6) | 1.58(1.23-2.03) | 0.0004^a^ | 1.01(0.76-1.35) | 0.94 |
| Coronary heart disease | 32(17.6) | 150(82.4) | 1.82(1.21-2.72) | 0.0037^a^ | 1.08(0.69-1.68) | 0.73 |
| Diabetes | 41(11.5) | 315(88.5) | 1.06(0.75-1.51) | 0.74 | 0.71(0.48-1.03) | 0.074 |
| Chronic obstructive pulmonary disease | 9(32.1) | 19(67.9) | 3.93(1.76-8.76) | 0.0008^a^ | 2.12(0.90-5.01) | 0.087 |
| Chronic kidney disease | 4(25.0) | 12(75.0) | 2.72(0.87-8.50) | 0.085 | 2.13(0.64-7.09) | 0.22 |
| Cerebrovascular disease | 18(24.7) | 55(75.3) | 2.76(1.60-4.77) | 0.0003^a^ | 1.69(0.94-3.04) | 0.082 |
| Hepatitis | 5(12.8) | 34(87.2) | 1.19(0.46-3.08) | 0.71 | 1.25(0.44-3.54) | 0.67 |
| Tuberculosis | 9(19.2) | 38(80.9) | 1.95(0.93-4.07) | 0.077 | 1.55(0.69-3.44) | 0.29 |
| Tumor | 32(29.4) | 77(70.6) | 3.66(2.37-5.63) | <0.0001^a^ | 3.39(2.12-5.42) | <0.0001^a^ |

^a^Significant at *P* < 0.05.

Table S2. Baseline characteristics of cancer patients and non-cancer controls (Before PS Matching vs. After PS Matching)

| **Characteristics** | **Before PS Matching** | | | **After PS Matching^c^** | | |
| --- | --- | --- | --- | --- | --- | --- |
|  | **Cancer**  **(n=109)** | **Non-cancer**  **(n=2556)** | ***P* value^b^** | **Cancer**  **(n=109)** | **Non-cancer (n=327)** | ***P* value^b^** |
| **Sex (n[%])** |  |  | 0.19 |  |  | 0.15 |
| Male | 61(56.0) | 1267(49.6) |  | 61(56.0) | 157(48.0) |  |
| Female | 48(44.0) | 1289(50.4) |  | 48(44.0) | 170(52.0) |  |
| **Age ( y- mean[SD])** | 61.7 (16.1) | 57.9 (15.9) | 0.015^a^ | 61.7 (16.1) | 61.6(16.0) | 0.98 |
| **Comorbidities (n[%])** |  |  |  |  |  |  |
| Hypertension | 30 (27.5) | 742 (29.0) | 0.73 | 30 (27.5) | 88(26.9) | 0.90 |
| Coronary heart disease | 11 (10.1) | 171 ( 6.7) | 0.17 | 11 (10.1) | 28(8.6) | 0.63 |
| Diabetes | 11 (10.1) | 345 (13.5) | 0.31 | 11 (10.1) | 40(12.2) | 0.55 |
| Chronic obstructive pulmonary disease | 0(0.0) | 28(0.0) | 0.63 | 0(0.0) | 5(1.5) | 0.34 |
| Chronic kidney disease | 0(0.0) | 16(0.6) | 1.00 | 0(0.0) | 3(0.9) | 0.58 |
| Cerebrovascular disease | 1(0.9) | 72(2.8) | 0.37 | 1(0.9) | 7(2.1) | 0.69 |
| Hepatitis | 0(0.0) | 39(1.5) | 0.41 | 0(0.0) | 5(1.5) | 0.34 |
| Tuberculosis | 1(0.9) | 46(1.8) | 1.00 | 1(0.2) | 7(2.1) | 0.69 |
| **Vital signs on admission** |  |  |  |  |  |  |
| Respiratory rate (times/min- mean[SD]) | 21.6(4.0) | 21.4(4.2) | 0.60 | 21.6(4.0) | 21.4(4.3) | 0.73 |
| Pulse (times/min- mean[SD]) | 91.5(16.7) | 91.3(15.7) | 0.86 | 91.5(16.7) | 89.3(15.9) | 0.23 |
| Temperature (℃- mean[SD]) | 36.9(0.8) | 36.8(0.8) | 0.44 | 36.9(0.8) | 36.8(0.7) | 0.24 |
| Pulse pressure (mmHg- mean[SD]) | 51.5(18.1) | 49.7(14.7) | 0.24 | 51.5(18.1) | 51.6(15.7) | 0.97 |
| Body mass index(kg/m^2^- mean[SD]) | 23.2(4.3) | 23.7(3.1) | 0.39 | 23.2(4.3) | 23.6(3.3) | 0.55 |
| Systolic pressure (mmHg- mean[SD]) | 130.7(20.4) | 130.7(19.0) | 0.99 | 130.7(20.4) | 133.1(19.4) | 0.29 |
| [Diastolic](link:diastolic) [pressure](link:pressure) (mmHg- mean[SD]) | 78.9(13.2) | 81.1(12.5) | 0.088 | 78.9(13.2) | 81.5(11.5) | 0.055 |
| **The signs and symptoms (n[%])** |  |  |  |  |  |  |
| Fever | 74(67.9) | 1777(69.5) | 0.72 | 74(67.9) | 235(71.9) | 0.43 |
| Fatigue | 13(11.9) | 382(15.0) | 0.39 | 13(11.9) | 59(18.0) | 0.14 |
| Cough | 53(48.6) | 1418(55.5) | 0.16 | 53(48.6) | 183(56.0) | 0.18 |
| Expectoration | 42(38.5) | 1031(40.3) | 0.71 | 42(38.5) | 137(41.9) | 0.54 |
| Dyspnea | 43(39.5) | 812(31.8) | 0.093 | 43(39.5) | 104(31.8) | 0.14 |
| Headache | 2(1.8) | 68(2.7) | 1.00 | 2(1.8) | 11(3.4) | 0.53 |
| Dizziness | 5(4.6) | 135(5.3) | 0.75 | 5(4.6) | 23(7.0) | 0.37 |
| Diarrhea | 20(18.4) | 495(19.4) | 0.79 | 20(18.4) | 64(19.6) | 0.78 |
| Thoracodynia | 18(16.5) | 379(14.8) | 0.63 | 18(16.5) | 42(12.8) | 0.34 |
| Nausea | 2(1.8) | 132(5.2) | 0.12 | 2(1.8) | 18(5.5) | 0.11 |
| Myalgia | 5(4.6) | 196(7.7) | 0.23 | 5(4.6) | 30(9.2) | 0.13 |
| Chills | 5(4.6) | 238(9.3) | 0.093 | 5(4.6) | 32(9.8) | 0.092 |
| Pharyngalgia | 4(3.7) | 132(5.2) | 0.49 | 4(3.7) | 15(4.6) | 0.79 |
| Vomiting | 2(1.8) | 73(2.9) | 0.77 | 2(1.8) | 11(3.4) | 0.53 |
| Abdominal pain | 2(1.8) | 27(1.1) | 0.33 | 2(1.8) | 7(2.1) | 1.00 |
| **Time from onset of symptom to** |  |  |  |  |  |  |
| Death (d- median[IQR]) | 18.8(10.7-33.2) | 20.0(14.3-28.8) | 0.56 | 18.8(10.7-33.2) | 18.6(13.3-27.2) | 0.93 |
| Discharged (d- median[IQR]) | 59.0(52.0-65.0) | 59.0(53.0-63.0) | 0.90 | 59.0(52.0-65.0) | 58.0(52.0-63.0) | 0.57 |

^a^Significant at *P* < 0.05.

^b^Calculated using the *χ^2^* test- Fisher’s exact test- *t* test or Mann-Whitney U test.

**^c^**Matched controls based on age.
